# Knowledge, Anxiety, Fear, and Psychological Distress About COVID-19 Among University Students in the United Arab Emirates

**DOI:** 10.3389/fpsyt.2020.582189

**Published:** 2020-10-22

**Authors:** Coumaravelou Saravanan, Ibrahim Mahmoud, Wiam Elshami, Mohamed H. Taha

**Affiliations:** ^1^Department of Family & Community Medicine and Behavioral Sciences, College of Medicine, University of Sharjah, Sharjah, United Arab Emirates; ^2^Department of Medical Diagnostic Imaging, College of Health Sciences, University of Sharjah, Sharjah, United Arab Emirates; ^3^College of Medicine and Medical Education Centre, University of Sharjah, Sharjah, United Arab Emirates

**Keywords:** anxiety, fear, COVID-19, distress, knowledge, United Arab Emirates, students

## Abstract

**Background:** Fear of infection, the epidemic situation, unexpected lockdown, and implementation of online classes are most likely affecting the psychological well-being of students during the COVID-19 pandemic. Therefore, this study aims to assess the level of knowledge, anxiety, and psychological distress concerning COVID-19 and their association with fear, gender, age, history of mental illness, time spent reading about COVID-19, program of study, and type of dwelling among students in the United Arab Emirates (UAE).

**Methods and Materials:** In this cross-sectional study, 433 students participated in a web-based survey. These were students at the University of Sharjah, coming from all the emirates of the UAE. Demographic scale, COVID-19 knowledge, anxiety, fear, and psychological distress scales were used to screen these problems.

**Results:** Of the 433 students, 278 (64.2%) were male and 155 (35.8%) were female. Overall, 353 (81.5%) exhibited adequate knowledge of COVID-19. Sixty-nine (15.9%) of students were anxious and 221 (51%) were in psychological distress. Students who exhibited anxiety concerning COVID-19 anxiety (odds ratio [OR]: 2.98) and fear (OR: 1.27), and who spent more than 4 h reading about COVID-19 (OR: 11.20) were more psychologically distressed. Students with a history of mental illness showed adequate knowledge of COVID-19; however, they were more psychologically distressed (OR: 5.93). Older students were less likely to have psychological distress (OR: 0.87).

**Conclusion:** Students possess adequate knowledge concerning COVID-19; however, they are psychologically distressed. Age, dwelling status, history of mental illness, anxiety, and fear significantly predicted psychological distress. Frequent web-based workshops that include insight, guidance, online counseling, scheduled activity, and coping mechanisms for COVID-19 are highly recommended. The authors discuss the implications for future research and provide recommendations for students and educational institutions.

## Introduction

The spread of any infectious disease is associated with anxiety, fear, psychological distress, and other symptoms of mental illness (1, 2). The COVID-19 pandemic is a major health catastrophe, with more than 11,000,000 cases confirmed (3). In the United Arab Emirates (UAE), up until the first week of July 2020, 50,857 cases had been reported (4). The fear of the COVID-19 infection, unexpected lockdown, and sudden implementation of online classes may lead to stress, anxiety, and other emotional problems among students (5, 6). Students need more ways to adjust and adapt to this situation as the lack of coping mechanisms to manage fear and anxiety may lead to significant physical and psychological distress.

Knowledge of COVID-19 and related safety and preventive measures is imperative to prevent disease spread and psychological distress (7, 8). However, students who spend a lot of time reading and watching the news about COVID-19, especially on social media, may get confused and misinterpret the seriousness of COVID-19 (9, 10). A study conducted in the Jordan identified that students have adequate knowledge about COVID-19, but are reluctant to wear face masks (8). These students are more anxious and fearful of infections as they either have insufficient information or misinterpret information about COVID-19. Maarefvand et al. (11) found that knowledge about the prevention of COVID-19 was significantly associated with stress among the public in Iran, but not among the student population. Hence, lack of knowledge and misconceptions about COVID-19 may lead to psychological distress. Past studies have shown that male students exhibited more anxiety and stress (12, 13), whereas another study concluded that there were no significant gender differences in the level of anxiety and stress among the student population (14). It is not necessarily the case that students with medical and health science backgrounds have more knowledge and are less anxious than arts and science students. One study conducted overseas found no significant difference between medical and non-medical students on anxiety and knowledge (7). However, a study conducted in the Middle East showed that medical students have more knowledge about COVID-19 than non-medical students (15). No data is available about anxiety and psychological distress during the COVID-19 outbreak among students with and without a history of mental illness. Therefore, there is a need to study students' knowledge, anxiety, amount of time spent reading about COVID-19, history of mental illness, gender differences, and programs pursued as these relate to psychological distress among students during the COVID-19 pandemic.

Stress is simply the body's response to changes that create taxing demands (16). Psychological distress is an aversive, negative state in which coping and adaptation processes fail to return an organism to physiological and psychological homeostasis (17). A very few studies have identified the prevalence of stress among the student population overseas, but no study was available in the UAE and other Middle Eastern countries. In China, anxiety levels among students were higher than the average (18). In a previous study in the UAE, it was reported that almost half of students experienced anxiety levels ranging from mild to severe (15). Most of the students were anxious and fearful concerning COVID-19 infection (19). Al-Rabiaaha (20) identified a significant relationship between fear, anxiety, and stress but not psychological distress. In addition, past studies (5, 14) conducted overseas used generalized anxiety scales, depression, anxiety, and stress scales, and fear scales to screen for anxiety, fear, and stress during COVID-19, but these scales can also be used for non–COVID-19-related situations. Scales such as the Coronavirus Anxiety Scale and the Fear of COVID-19 Scale that exclusively measure anxiety and fear related to COVID-19 were not used among the student population. Using these scales to screen for anxiety and fear would most likely provide a more exact picture of COVID-9-related anxiety and fear. In addition, past studies did not measure psychological distress or its association with anxiety and fear. Educational institutions and the Ministry of Health and Prevention in the UAE provide adequate awareness about COVID-19. However, we are not sure of the level of knowledge, fear, and anxiety among students, and the association with psychological distress and no distress.

The University of Sharjah is one of the largest universities in the UAE, with students hailing from all the emirates. Conducting research on students from this university will represent students from other universities as well. Based on the above-mentioned past literature, this study aimed to assess the level of knowledge, anxiety, and psychological distress among students. Further, this study aimed to measure the differences and associations between knowledge, anxiety, psychological distress, and demographic characteristics such as gender, age, history of mental illness, time spent reading about COVID-19, program (course) of study, and type of dwelling place (villa, apartment, and dorm or shared apartment). In addition, this study aimed to measure to what extent lack of knowledge, anxiety, fear, gender, history of mental illness, program (course) of study, time spent reading about COVID-19, age of the participants, and dwelling place predicted the psychological distress caused by COVID-19. The first hypothesis of this study is that there would be a significant association and differences between knowledge, anxiety, fear, psychological distress, and demographic characteristics (age, gender, course, dwelling status, time spent reading about COVID-19, and history of mental illness). The second hypothesis of this study is that demographic characteristics, knowledge, anxiety, and fear are significant predictors for psychological distress.

## Method

### Participants

In this cross-sectional study, 433 students participated in a web-based survey. We collected data from students at the University of Sharjah (UOS) from May 1 to 30, 2020. The demographic information on the participants is shown in Table 1.

**Table 1:** Demographic characteristics of participants and their levels of knowledge, anxiety, and psychological distress.

**Variable**	**No. (%)**
Age, years Mean (SD)	21 (2.9)
Gender	
Male	278 (64.2)
Female	155 (35.8)
College	
Arts	200 (46.2)
Engineering	115 (26.5)
Health science/science	74 (17.1)
Medical	44 (10.2)
Dwelling status	
Villa	251 (58)
Apartment	140 (32.3)
Dorm/shared	42 (9.7)
Time spent reading about COVID-19	
<1 h	290 (67)
1–2 h	91 (21)
3–4 h	25 (5.8)
More than 4 h	27 (6.2)
History of mental illness	
No	363 (83.8)
Yes	70 (16.2)
Knowledge of COVID-19	
Mean (SD)	4.5 (1.2)
Inadequate knowledge	80 (18.5)
Adequate knowledge	353 (81.5)
COVID-19 anxiety	
Mean (SD)	5.4 (3.6)
No	364 (84.1)
Yes	69 (15.9)
Psychological distress	
Mean (SD)	15.8 (6)
No	212 (49)
Yes	221 (51)
COVID-19 fear Mean (SD)	16.6 (6.3)

*SD, standard deviation*.

### Sample Size

Based on the sample size calculation, with a 5% margin of error and 95% confidence interval, this study requires a minimum of 377 participants. However, this study expects 10% of questionnaires to be incomplete, so a minimum of 414 participants are needed.

### Procedure

Once the study had received ethical and research approval (REC-20-05-07-01) from the Ethics and Research Committee of the University of Sharjah, we sent an online invitation through the university's official portal to all UOS students. The online invitations highlighted that participation was voluntary, participants could withdraw at any time, and all the information collected would be kept confidential. Online consent was received from all the participants before they completed the questionnaire. Proper contact details were provided on the first page of the survey if participants wanted to clarify their doubts about this study. The online survey was set up to provide an automatic thank you statement to the students who completed the survey.

### Materials

#### Sociodemographic Scale

A sociodemographic scale was created for this study. The scale measured the participants' gender, course (program of study), age, history of mental illness, and dwelling status (villa, apartment, dorm or shared apartment).

#### Knowledge About COVID-19

A scale was developed to measure public knowledge about COVID-19. This scale consisted of six items measured on a five-point Likert scale. One point was given for correct answers, and no points were given for incorrect or uncertain answers. Participants with scores four and above were rated “adequate knowledge” and <4 indicated “inadequate knowledge” on COVID-19 (10). The alpha reliability value of this study was 0.79.

#### Coronavirus Anxiety Scale

The Coronavirus Anxiety Scale was used to measure anxiety about COVID-19. Each item was rated on a five-point Likert scale to reflect the frequency of the symptom, ranging from 0 (not at all) to 4 (nearly every day) over the preceding 2 weeks. Participants who scored nine and above were considered as experiencing COVID-19 anxiety and <9 indicated no COVID-19 anxiety. The reliability of this scale ranges from 81 to 87 (21). The alpha reliability value of this study was 0.88.

#### Fear of COVID-19 Scale

This scale measured the fear of COVID-19 infection. The participants indicated their level of agreement with statements using a five-item Likert-type scale. The minimum score possible for each question was one, and the maximum was five. The total score was calculated by adding the scores for each item (giving a total ranging from 7 to 35). The higher the score, the greater the fear of COVID-19. Reliability values are α = 0.82 for internal consistency and 0.72 on retest and test (22). The alpha reliability value of this study was 0.89.

#### The Kessler Psychological Distress Scale

The Kessler Psychological Distress Scale (K-6) is a shortened, six-item version of the K-10. This scale was used to measure psychological and non-psychological distress. It measures non-specific psychological distress based on a framework that includes behavioral, emotional, cognitive, and psychophysiological manifestations. In this study, responses were summed to produce a total score ranging from 6 to 30, with higher scores signifying more distress. Participants who scored 16 and above were considered to be experiencing psychological distress and a score of <16 was considered to indicate no psychological distress. Based on the cut-off score on this scale, this study analyzed psychological distress as a categorical variable (psychological distress and no psychological distress) and total score of the psychological distress scale is also considered as a continuous variable in the results. K-6 has been found to be reliable, with Cronbach's α ranging from 0.89 to 0.92 (23, 24). The alpha reliability value of this study is 0.88.

### Statistical Analysis

Frequencies with proportions and means with standard deviations (SD) were reported to describe the characteristics of the study participants and their levels of knowledge, anxiety, psychological distress, and fear.

All variables were analyzed in two ways. First, a chi square text (*X*^2^) was used for the categorical variables (adequate and inadequate knowledge, anxiety and no anxiety, psychological distress, and no psychological distress), as well as for other variables (gender, dwelling status, history of mental illness, and time spent reading about COVID-19) and counts (number) with percentages are presented in the tables. Second, a *t*-test and analysis of variance (ANOVA) were used for the continuous variables (knowledge, anxiety, fear, and psychological distress) and data presented as means (SD). The fear scale and age were analyzed as continuous variables throughout this study.

The primary outcome, the psychological distress scale, was sorted into two categories based on the cutoff score of the scale, where 16 and above indicated participants experienced psychological distress (Yes) and below 16 indicated participants did not experience psychological distress (No). Variables that were significantly associated with psychological distress in bivariate analyses were included in the multivariate binary logistic regression model. Age and fear were included as continuous variables, and dwelling status, time spent reading about COVID-19, history of mental illness, and anxiety were included as categorical variables in the binary logical regression model.

The Omnibus Tests of Model Coefficients showed that the binary logistic regression models were statistically significant. The case-wise plots were not produced because no outliers were found, and no multicollinearity detected (variance inflation factor <3). Statistical significance was set at *p* ≤ 0.05. The data analyses were performed with the IBM Statistical Package for Social Sciences (SPSS) Statistics for Windows Version 25.0 (25).

## Results

Of the 433 study participants, 278 (64.2%) were men. The mean age of the study participants was 21 years (SD ± 2.9 years, range 18–38 years). Table 1 shows the characteristics of the study participants.

### Prevalence of Knowledge, Anxiety, and Psychological Distress

Table 1 shows that the prevalence of students who were not knowledgeable about COVID-19 was 80 (18.5%) and knowledgeable 353 (81.5%). There were 364 (84.1%) students who showed no anxiety and 69 (15.9%) who showed anxiety. Prevalence among students of no psychological distress was 212 (49%) and psychological distress 221 (51%).

### Association and Difference Between Demographic Variables and Knowledge About COVID-19

Table 2 shows that there were no significant differences in the level of knowledge based on age, gender, educational program, and dwelling status (*p* > 0.05). However, participants with a history of mental illness showed a higher level of knowledge than participants without a history of mental illness (92.9 vs. 79.3%, *p* = 0.002). Furthermore, participants with lower levels of fear of COVID-19 had more knowledge than participants with higher levels of fear of COVID-19 (mean 16.3 ± 5.8 vs. 18.1 ± 7.8, *t* = 2.40, *p* = 0.017).

**Table 2:** Association and differences between knowledge and demographic variables, psychological distress, anxiety, fear.

**Variable**	**Knowledge of COVID-19 status**						
	***χ^2^*-test**					***t*****-test/ANOVA**	
	**Inadequate**	**Adequate**	***χ^2^***	***P***	**M(SD)**	**t/F**	***P***
	**knowledge**	**knowledge**					
	***n* (%)**	***n* (%)**					
^*^Age, years							
Mean (SD)	21.8 (3.3)	21.3 (2.8)		0.218		1.23	
Gender							
Male	48 (17.3)	230 (82.7)	0.8	0.385	4.6 (1.2)	−1.77	0.077
Female	32 (20.6)	123 (79.4)			4.4 (1.2)		
Educational program							
Arts	38 (19)	162 (81)	2.3	0.522	4.4 (1.2)	1.71	0.165
Engineering	25 (21.7)	90 (78.3)			4.5 (1.1)		
Health sciences/sciences	10 (13.5)	64 (86.5)			4.7 (1.2)		
Medical	7 (15.9)	37 (84.1)			4.8 (1.3)		
Dwelling status							
Villa	42 (16.7)	209 (83.3)	1.5	0.469	4.1 (1.2)	2.77	0.064
Apartment	28 (20)	112 (80)			4.4 (1.1)		
Dorm/shared	10 (23.8)	32 (76.2)			4.2 (1.4)		
Time spend reading about Covid-19							
<1 h	55 (19)	235 (81)	3.2	0.356	4.5 (1.2)	1.81	0.145
1–2 h	12 (13.2)	79 (86.8)			4.7 (1.1)		
3–4 h	6 (24)	19 (76)			4.3 (0.9)		
More than 4 h	7 (25.9)	20 (74.1)			4.3 (1.4)		
History of mental illness							
No	75 (20.7)	288 (79.3)	7.1	**0.008**	4.4(1.2)	−3.18	**0.002**
Yes	5 (7.1)	65 (92.9)			4.9 (1)		
COVID-19 anxiety							
No	64 (17.6)	300 (82.4)	1.2	0.271	4.5 (1.2)	1.07	0.285
Yes	16 (23.2)	53 (76.8)			4.4 (1.4)		
Psychological distress							
No	35 (16.5)	177 (83.5)	1.1	0.302	4.6 (1.2)	0.83	0.409
Yes	80 (18.5)	176 (79.6)			4.5 (1.2)		
COVID-19 fear							
Mean (SD)	18.1 (7.8)	16.3 (5.8)		**0.017**		2.40	

* *Analyzed as continuous variable (t-test) only, P-values in bold are statistically significant. M= Mean, SD= standard deviation*.

### Association and Difference Between Demographic Variables and COVID-19 Anxiety

Table 3 shows that male participants (18%), living in a dorm or shared apartment (26.2%), who spent more than 4 h reading about COVID-19 (44.4%), with a history of mental illness (35.7%), with psychological distress (27.6%), and more fear of COVID-19 (23 ± 6.9) have higher levels of anxiety than women (12.3%), living in a villa (15%), who spent <1 h reading about COVID-19 (8%), without a history of mental illness (12%), without psychological distress (4%), and less fear of COVID-19 (15.4 ± 5.4), *p* ≤ 0.05.

**Table 3:** Association and differences between anxiety and demographic variables, knowledge, psychological distress, fear.

**Variable**	**COVID-19 anxiety status**						
	***χ^2^*-test**					***t*****-test/ANOVA**	
	**No *N* (%)**	**Yes *N* (%)**	***χ^2^***	***P***	**M(SD)**	**t/F**	***P***
Age, years^*^							
Mean (SD)	21.8 (3.3)	21.3 (2.8)		0.397		0.85	
Gender							
Male	228 (82)	50 (18)	2.4	0.119	6.3 (3.2)	−7.49	**0.0001**
Female	136 (87.7)	19 (12.3)			3.8 (3.8)		
Educational program							
Arts	166 (83)	34 (17)	4.8	0.189	5.9 (3.4)	3.27	**0.021**
Engineering	102 (88.7)	13 (11.3)			4.8 (3.4)		
Health sciences/sciences	63 (85.1)	11 (14.9)			5.5 (4)		
Medical	33 (75)	11 (25)			4.5 (4.3)		
Dwelling status							
Villa	213 (84.9)	38 (15.1)	3.7	0.157	5.7 (3.2)	5.82	**0.003**
Apartment	120 (85.7)	20 (14.3)			4.6 (3.9)		
Dorm/shared	31 (73.8)	11 (26.2)			6.5 (4.5)		
Time spend reading about COVID-19							
<1 h	267 (92.1)	23 (7.9)	48.5	**0.0001**	4.5 (3.1)	25.0	**0.0001**
1–2 h	67 (73.6)	24 (26.4)			6.8 (3.4)		
3–4 h	15 (60)	10 (40)			7.2 (4.9)		
More than 4 h	15 (55.6)	12 (44.4)			9 (4.5)		
History of mental illness							
No	319 (87.9)	44 (12.1)	24.4	**0.0001**	5.2 (3.5)	−3.37	**0.001**
Yes	45 (64.3)	25 (35.7)			6.7 (4)		
COVID-19 knowledge							
Inadequate knowledge	64 (80)	16 20)			5.4 (4.4)		
			1.21	0.270		−0.15	0.878
Adequate							
knowledge	300 (85)	53 (15)			5.4 (3.4)		
Psychological distress							
No	204 (96.2)	8 (3.8)	45.9	**0.0001**	14.9 (5.7)	−7.06	**0.0001**
Yes	160 (72.4)	61 (27.6)			20.7 (4.9)		
COVID-19 fear^*^							
Mean (SD)	15.4 (5.4)	23 (6.9)		**0.0001**		−10.2	

* *Analyzed as continuous variable (t-test) only, P-values in bold are statistically significant. M, Mean; SD, standard deviation*.

### Association and Difference Between Demographic Variables and Psychological Distress

Table 4 shows that participants who are younger (21.1 ± 2.5), living in apartments (59.3%), spend more than 4 h reading about COVID-19 (92.6%), with a history of mental illness (83%), with anxiety (88.4%), and more fear of COVID-19 (19.7 ± 6) have higher levels of psychological distress than older (21.7 ± 3.3) participants, living in villas (45.4%), who spend <1 h reading about COVID-19 (42.8%), without a history of mental illness (45%), without anxiety (44%), and less fear of COVID-19 (13.3 ± 4.7), *p* < 0.05.

**Table 4:** Association and differences between psychological distress and demographic variables, knowledge, anxiety, fear.

**Variable**	**Psychological distress status**						
	***χ^2^*-test**					***t*****-test/ ANOVA**	
	**No *n* (%)**	**Yes *n* (%)**	***χ^2^***	***P*-value**	**M(SD)**	**t/F**	***P***
Age, years^*^							
Mean (SD)	21.7 (3.3)	21.1 (2.5)		**0.019**		2.36	
Gender							
Male	134 (48.2)	144 (51.8)	0.2	0.672	16 (6)	−0.55	0.580
Female	78 (50.3)	77 (49.7)			15.6 (5.9)		
Educational program							
Arts	101 (50.5)	99 (49.5)	3.1	0.382	15.6 (6)	1.40	0.332
Engineering	61 (53)	54 (47)			15.3 (5.7)		
Health sciences/sciences	32 (43.2)	42 (56.8)			16.3 (6.1)		
Medical	18 (40.9)	26 (59.1)			17.1 (6.3)		
Dwelling status							
Villa	137 (54.6)	114 (45.4)	7.6	**0.022**	15.3 (6.1)	2.57	**0.053**
Apartment	57 (40.7)	83 (59.3)			16.6 (5.6)		
Dorm/shared	18 (42.9)	24 (57.1)			16.6 (6)		
Time spend reading about COVID-19							
<1 h	166 (57.2)	124 (42.8)	36.4	**0.0001**	14.7 (5.9)	13.54	**0.002**
1–2 h	39 (42.9)	52 (57.1)			17.1 (5.7)		
3–4 h	5 (20)	20 (80)			18.6 (5)		
More than 4 h	2 (7.4)	25 (92.6)			20.6 (4.4)		
History of mental illness							
No	200 (55.1)	163 (44.9)	33.8	**0.0001**	15 (5.7)	−6.65	**0.0001**
Yes	12 (17.1)	58 (82.9)			19.9 (5.5)		
COVID-19 knowledge							
Inadequate knowledge	35 (43.8)	45 (56.3)	1.1	0.302	16.1 (5.3)	0.49	0.626
Adequate knowledge	177 (50.1)	49 (49.9)			15.7 (6.1)		
COVID-19 anxiety							
No	204 (56)	160 (44)	45.9	**0.0001**	14.9 (5.7)	−7.87	**0.0001**
Yes	8 (11.6)	61 (88.4)			20.7 (4.5)		
COVID-19 fear^*^							
Mean (SD)	13.3 (4.7)	19.7 (6)		**0.0001**		−12.15	

* *Analyzed as continuous variable (t-test) only, P-values in bold are statistically significant. M, Mean; SD, standard deviation*.

Table 5 shows the correlations between knowledge and anxiety (*r* = 0.026, *p* = 0.584), knowledge and fear (*r* = −0.098, *p* = 0.043), knowledge and psychological distress (*r* = −0.013, *p* = 0.794), anxiety and fear (*r* = −0.481, *p* < 0.0001), anxiety and psychological distress (*r* = −0.375, *p* < 0.0001), and fear and psychological distress (*r* = −0.494, *p* < 0.0001).

**Table 5:** Correlations among knowledge, anxiety, fear and psychological distress.

**Variables**		**1**	**2**	**3**	**4**
1	COVID-19 knowledge	–			
2	COVID-19 anxiety	0.026	–		
3	COVID-19 fear	−0.098^*^	0.481^**^	–	
4	Psychological distress	−0.013	0.375^**^	0.494^**^	–

* *Correlation is significant at the 0.05 level*;

** *Correlation is significant at the 0.01 level*.

### Predictors of Psychological Distress

Variables that are significantly associated (age, dwelling status, time spent reading about COVID-19, history of mental illness, anxiety, and fear) with psychological distress (Table 4) were included in the multivariate binary logistic regression model. Table 6 demonstrates the multivariate binary logistic regression for the predictors of psychological distress among the study participants. Our regression model explained only 45% of the variability in psychological distress, controlling for age, dwelling status, time spent on reading about COVID-19, history of mental illness, anxiety, and fear. Older participants [odds ratios: (OR) 0.87, 95% confidence interval: (CI) 0.80–0.95, *p* = 0.002] are less likely to suffer from psychological distress compared to younger participants. Furthermore, participants who are living in apartments (OR= 2.48, 95% CI: 1.44–4.24, *p* = 0.001), spent more than 4 h reading about COVID-19 (OR= 11.20, 95% CI: 2.23–56.24, *p* = 0.003), have a history of mental illness (OR= 5.93, 95% CI: 2.66–13.26, *p* < 0.0001), have anxiety (OR= 2.98, 95% CI: 1.18–7.50, *p* < 0.021), and have more fear of COVID-19 (OR= 1.27, 95% CI: 1.20–1.34, *p* < 0.0001) are more likely to suffer from psychological distress compared to participants living in a villa, who spent <1 h reading about COVID-19, without a history of mental illness and anxiety, and who have less fear of COVID-19.

**Table 6:** Predictors of the psychological distress among study participants.

**Variable**	**OR**	**95% CI**	***P*-value**
Age	0.87	0.80–0.95	**0.002**
Dwelling status			
Villa	1		
Apartment	2.48	1.44–4.24	**0.001**
Dorm/shared	1.55	0.65–3.73	0.326
Time spend reading about COVID-19			
<1 h	1		
1–2 h	0.72	0.39–1.32	0.284
3–4 h	1.43	0.41–5.00	0.577
More than 4 h	11.20	2.23–56.24	**0.003**
History of mental illness			
No	1		
Yes	5.93	2.66–13.26	**0.0001**
COVID-19 anxiety			
No	1		
Yes	2.98	1.18–7.50	**0.021**
COVID-19 fear	1.27	1.20–1.34	**0.0001**

*P-values in bold are statistically significant. OR, Odds Ratios; CI, Confidence Interval*.

## Discussion

In this study, 81.8% of students show adequate knowledge about COVID-19. The prevalence of anxiety is 16% and the psychological distress is 51%. History of mental illness and COVID-19 fear are significantly associated with knowledge, anxiety, and psychological distress. Furthermore, dwelling status and time spent on reading and watching about COVID-19 are associated with anxiety and psychological distress. However, gender and educational program are associated with anxiety only. Living in apartments, spending more than 4 h reading and watching about COVID-19, a history of mental illness, and COVID-19 anxiety are significantly predicting psychological distress.

Similar to the present study students in Jordan (7), Iran (19), Italy (26), and the Philippines (9) also expressed adequate knowledge about COVID-19. The study showed that most of the educational institutions had provided adequate information about COVID-19. This could be the reason for students to have adequate knowledge about the mode of transmission of COVID-19 and preventive measures, but some students who are dependent on social media may end up with incorrect information about COVID-19 (8, 27). In this study male students were found to be more knowledgeable than female students. However, a study conducted in Jordan found that females had more knowledge about COVID-19 than males (12). Studies found that students have adequate knowledge about safety measures such as hand sanitizing, avoiding social gatherings, and mode of transmission of infection, but some students were reluctant to wear masks (8).

Medical students have more knowledge of COVID-19, followed by students in the health sciences, engineering, and arts programs. The result is consistent with another study conducted in the UAE (15). However, a study conducted in Jordan found no significant difference between medical and non-medical students on knowledge about COVID-19 (7). The result of this study is consistent with a study conducted in Russia, which found no significant relationship between knowledge and the amount of time spent on reading and watching about COVID-19 (28). These results show that time spent reading about COVID-19 is not significantly associated with knowledge, but the amount of scientific knowledge students have about COVID-19 is imperative for the adherence to safety procedures. COVID-19 is an unprecedented situation that requires a great deal of adaptation and assimilation.

The first hypothesis of this study, that participants who have a history of mental illness show adequate knowledge about COVID-19, is fully accepted. However, a study conducted among the public in China found that people with a history of mental illness were prone to complications of COVID-19, due to poor insight and difficulties adhering to safety measures (29, 30). In addition, this study found no significant association between knowledge and psychological distress, and knowledge was not a significant predictor for psychological distress. Past studies had identified that lack of knowledge, fear of infection, and worries about COVID-19 most likely trigger stress (7, 8, 11).

In this study, the prevalence of anxiety was 16%, less than was found in Spain-−22. 3% (5), China–22.1% (14), Jordan-−22.5% (31), and the Kingdom of Saudi Arabia-−23% (20). The mean value of this study result is also less than a study conducted in India (32). All the earlier studies had used the Generalized Anxiety Questionnaire to screen for anxiety, but this study used the Coronavirus Anxiety Scale, which exclusively measured anxiety about COVID-19. Students who were anxious experienced more fear of COVID-19. Only one study measured the fear of COVID-19, and that study used a general fear scale to assess fear during COVID-19 (20), but this study used the COVID-19 Fear Scale, which is exclusively used to measure fear of COVID-19. This result of this study shows less coronavirus anxiety than other studies conducted among the student population overseas. Students are well-informed about the COVID-19 safety and precaution measures in the UAE, which could be another reason for less anxiety found in this study. The result of this study is consistent with the study conducted in China that male students were significantly more anxious than female students (13), but females experienced more anxiety than males in Jordan (31). No gender difference was found in another study conducted in China (14). The differences in gender concerning anxiety were due to the nature and time of the study.

The result of this study is consistent with a study conducted in China that Arts students experienced more anxiety than students in other program (13). The study shows that educational institutions need to provide more awareness about COVID-19 among students pursuing Arts programs, which may further reduce anxiety about COVID-19. No studies have been conducted on the association between dwelling status and anxiety, but this study has found that students staying in a shared house or dorm (hostel) are more anxious about COVID-19 than those staying in a villa or apartment. Students who were anxious were often worried about the spread of COVID-19 and viral infection for themselves and their families (9). In this study students with a history of mental illness were significantly more anxious and stressful than those who did not have a history of mental illness. During COVID-19, most countries have implemented lockdown and suggested online classes, and this may impact students with no history of mental illness, but would most likely affect students with a history of mental illness (6). Students with a history of mental illness, therefore, need to consult a mental health professional to protect themselves from COVID-19 anxiety, fear, and psychological distress.

In this study, the prevalence of psychological distress is higher (51%) compared to students in Spain 28.14% (5) during COVID-19. The major cause of psychological distress could be due to sudden lockdown, shifting from traditional classes to online classes, no social gathering, and fear and anxiety about COVID-19. Students who spend more than 4 h reading and watching information about COVID-19 are more anxious and psychologically distressed than those who spend less time on COVID-19. A study conducted in China found that health-care workers and younger people who spent more than 3 h reading and watching information about COVID-19 were more anxious than those who spent <2 h (10). This study recommends that students and the public reduce their time spent reading and watching about COVID-19 on social media as this may affect their anxiety and psychological distress levels. However, during lockdown, students are more engaged in social media, especially reading about COVID-19 (8, 26). Therefore, educational institutions can provide non-educational and educational online activities to engage students in useful activities.

The second hypothesis of this study, that staying in an apartment, age of participant, more than 4 h spent read about COVID-19, history of mental illness, anxiety, and fear significantly predict psychological distress, is partially accepted. The result of this study is partially consistent with other studies conducted overseas that anxiety and fear were associated with stress, but not with psychological distress (20, 32). This study regression model predicted only 45% of the variability on psychological distress. This indicate that there are other influential factors affecting psychological distress during COVID-19 were not captured by our study.

This is the first study that has found that the history of mental illness predicts psychological distress among the student population during COVID-19. Students who are staying in shared housing experienced more psychological distress as they may think that they are susceptible to COVID-19. Therefore, treating anxiety, fear, and the history of mental illness will most likely reduce the psychological distress among students during COVID-19. Students who experience higher psychological distress are more prone to substance abuse, insomnia, suicidal behavior, poor academic performance, and lack of concentration and attention (33–35). Hence, educational institutions need to be aware of the associated factors and consequences of psychological distress among students during COVID-19. Concurrently, students who think that they are experiencing or are prone to psychological problems should consult a mental health professional or student counselor to prevent the consequences of their illness.

### Implications

During lockdown, students should find an alternative way to engage themselves usefully instead of reading excessively about COVID-19 as this study result shows that spending more than 4 h reading about COVID-19 induces anxiety. Because the UAE government has opened most of the online apps for free communication, students could do online chatting and indulge in online group games with their friends. This would reduce excessive time spent reading about COVID-19. In this pandemic situation, students without a history of mental illness find difficulties coping with this unexpected situation, as do students with mental illness. However, students with a history of mental illness need to be more cautious as they are mentally more vulnerable to the aggravation of their mental illness during COVID-19. We suggest that educational institutions should provide some additional supportive counseling to students who have a history of mental illness as this study result shows that they are more prone to experiencing psychological distress.

Educational institutions need to provide more insight about COVID-19 among non-medical students, such as those in the arts and sciences, as they have less knowledge and are more anxious about COVID-19. We are aware that most of the educational institutions provide adequate awareness. However, conducting a frequent web-based question and answer session might help students to manage their doubts about COVID-19 and also alleviate their anxiety and fear related to COVID-19.

### Limitation

First, this study data was collected using a convenience sampling method, and this may lead to sampling bias. The study data was collected from one university, but this university is one of the largest higher education institutions in the UAE, and students come from all the emirates and other Gulf countries. During the data collection period, most of the students had returned to their homes because of the lockdown. Hence, the sample may be considered representative of university students who live in the UAE. Third, if this study had been conducted before May 2020, the results may have been different, as at the beginning of the spread of COVID-19 students may have been more anxious and psychologically distressed. Last, this study used self-assessment questionnaires, which may lead to a biased result. Students who exhibited anxiety and psychological distress in the questionnaire were not interviewed clinically to confirm their diagnosis. However, all the studies conducted during COVID-19 had used self-assessment scale to screen the psychological problems.

### Recommendations for Future Research

So far, all the studies among the student population have focused on domestic students, but not on international students. Therefore, measuring international students' anxiety, fear, and psychological distress would be beneficial to know their psychological well-being status. Based on the results of this study, we recommend researchers use the COVID-19 anxiety and fear scales to assess anxiety and fear during COVID-19 rather than using general anxiety and fear scales as these may not evaluate COVID-19 anxiety and fear specifically. Screening for psychological distress and its predictors among the public would be beneficial. A similar study on a larger sample would be beneficial to allow comparison with the results of this study.

### Conclusion

Overall, students have sufficient knowledge of COVID-19. Students in this study were found to have slightly less anxiety and fear of COVID-19 than was found in studies conducted overseas, while psychological distress was higher. Students studying arts and sciences are experiencing more psychological distress than students studying in medical and health science programs. Age, dwelling status, history of mental illness, anxiety, and fear significantly predicted psychological distress. Educational institutions need to provide academic and professional counseling to students to reduce their psychological distress and improve their academic performance.

## Data Availability Statement

The datasets used and/or analyzed during the current study are available from the corresponding author on reasonable request.

## Ethics Statement

The studies involving human participants were reviewed and approved by Ethics and Research committee (REC-20-05-07-01) of the University of Sharjah. All the participants provided their online consent to participate in this study.

## Author Contributions

CS wrote the discussion section and arranged the manuscript. IM wrote the result section. WE and MT wrote the introduction section. All authors reviewed the manuscript and approved it. All authors contributed to the article and approved the submitted version.

## Conflict of Interest

The authors declare that the research was conducted in the absence of any commercial or financial relationships that could be construed as a potential conflict of interest.
